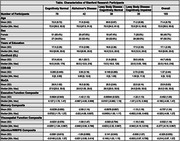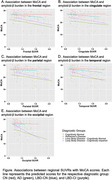# Occipital amyloid‐β burden and clinical correlates in Lewy body disease

**DOI:** 10.1002/alz.093253

**Published:** 2025-01-09

**Authors:** Hillary Vossler, Carla Abdelnour, Christina B. Young, Joseph R. Winer, Alena Smith, Viktorija Smith, Marian Shahid, Edward N. Wilson, Guido A. Davidzon, Elizabeth Mormino, Kathleen L. Poston

**Affiliations:** ^1^ Stanford University School of Medicine, Stanford, CA USA; ^2^ Stanford University, Stanford, CA USA

## Abstract

**Background:**

Accurate assessment of amyloid‐β burden in Lewy body disease (LBD) is crucial due to the frequent co‐occurrence of amyloidosis. Current amyloid‐β PET imaging protocols often overlook the occipital lobe, a relevant area in Lewy body disease (LBD). We aimed to determine whether regional amyloid‐β in LBD is associated with global cognition, daily functioning, and neuropsychological performance.

**Method:**

We included 193 Stanford research participants: 78 Cognitively Normal (CN), 42 Alzheimer’s disease (AD), 38 LBD‐Cognitively Normal (LBD‐CN), and 35 LBD‐Cognitively Impaired (LBD‐CI) who underwent 18F‐Florbetaben PET imaging (Table). We calculated SUVRs for the frontal, cingulate, parietal, temporal, and occipital regions using the cerebellum as the reference region. We then used linear regression models adjusted for age, sex, and education to examine the association between regional amyloid‐β burden and global cognition (measured with MoCA), daily functioning (measured with CDR‐SB), and performance in memory, executive function, visuospatial function, and attention/working memory/processing speed (measured with composite scores).

**Result:**

In the LBD‐CI group, amyloid‐β burden in all regions, except the cingulate, was associated with worse MoCA (β = ‐14.31 (SE = 3.59), p < 0.0001) (Figure) and CDR‐SB scores (β = 5.05 (SE = 1.71), p < 0.004). Similarly, amyloid‐β burden in the frontal (β = ‐1.27 (SE = 0.60), p < 0.05), cingulate (β = ‐1.24 (SE = 0.59), p < 0.05), parietal (β = ‐1.42 (SE = 0.62), p < 0.05), and temporal (β = ‐1.68 (SE = 0.70), p < 0.05) regions was associated with worse memory performance, whereas amyloid‐β burden in the occipital region (β = ‐1.76 (SE = 0.88), p < 0.05) was associated with worse executive function. However, only the association between occipital amyloid‐β burden and MoCA scores survived multiple comparison correction. In the LBD‐CN group, regional amyloid‐β burden showed no associations with clinical outcomes.

**Conclusion:**

Our study suggests the potential significance of the occipital region in assessing amyloid‐β burden among individuals with cognitive impairment due to LBD, as it is associated with worse global cognition. Further validation studies should include the occipital region when calculating SUVRs should be considered in people with LBD.